# The Impact of the C-Terminal Domain on the Interaction of Human DNA Topoisomerase II α and β with DNA

**DOI:** 10.1371/journal.pone.0014693

**Published:** 2011-02-16

**Authors:** Kathryn L. Gilroy, Caroline A. Austin

**Affiliations:** Institute for Cell and Molecular Biosciences, University of Newcastle Upon Tyne, Newcastle upon Tyne, United Kingdom; University Paris Diderot-Paris 7, France

## Abstract

**Background:**

Type II DNA topoisomerases are essential, ubiquitous enzymes that act to relieve topological problems arising in DNA from normal cellular activity. Their mechanism of action involves the ATP-dependent transport of one DNA duplex through a transient break in a second DNA duplex; metal ions are essential for strand passage. Humans have two isoforms, topoisomerase IIα and topoisomerase IIβ, that have distinct roles in the cell. The C-terminal domain has been linked to isoform specific differences in activity and DNA interaction.

**Methodology/Principal Findings:**

We have investigated the role of the C-terminal domain in the binding of human topoisomerase IIα and topoisomerase IIβ to DNA in fluorescence anisotropy assays using full length and C-terminally truncated enzymes. We find that the C-terminal domain of topoisomerase IIβ but not topoisomerase IIα affects the binding of the enzyme to the DNA. The presence of metal ions has no effect on DNA binding. Additionally, we have examined strand passage of the full length and truncated enzymes in the presence of a number of supporting metal ions and find that there is no difference in relative decatenation between isoforms. We find that calcium and manganese, in addition to magnesium, can support strand passage by the human topoisomerase II enzymes.

**Conclusions/Significance:**

The C-terminal domain of topoisomerase IIβ, but not that of topoisomerase IIα, alters the enzyme's K_D_ for DNA binding. This is consistent with previous data and may be related to the differential modes of action of the two isoforms *in vivo*. We also show strand passage with different supporting metal ions for human topoisomerase IIα or topoisomerase IIβ, either full length or C-terminally truncated. They all show the same preferences, whereby Mg > Ca > Mn.

## Introduction

**Figure 1:**
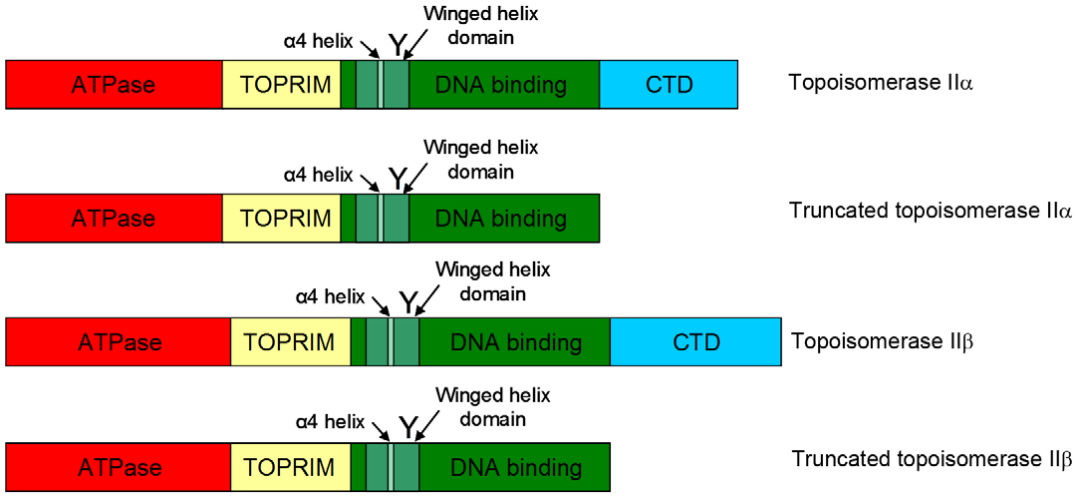
Schematic showing the domains of full length and truncated topoisomerase II isoforms. ‘Y’ is the active site tyrosine, within the winged helix domain.

Type II DNA topoisomerases are ubiquitous enzymes that are essential for cellular survival, acting to relieve torsional stress arising in DNA from normal cellular activity such as replication and transcription. Their mechanism of action involves the ATP-dependent transport of one DNA duplex through a transient break in a second DNA duplex. Humans have two isoforms, topoisomerase IIα and topoisomerase IIβ, which are encoded on different chromosomes and have distinct cellular roles. Human topoisomerase IIα is required for chromosome segregation, while topoisomerase IIβ plays a key role in the regulation of transcription [1]–[6] and is crucial for the late stages of neuronal development [7]. Topoisomerase IIα and IIβ are 68% identical in amino acid sequence and share a similar domain structure that is comparable to other type II enzymes. They have an N-terminal ATPase domain, a central core domain housing the active site tyrosine required for DNA cleavage and a C-terminal domain that becomes post-translationally modified. While the core domain from type II topoisomerases from yeast and bacteria has been crystallized and the structure solved, and the ATPase domain has been crystallized from bacterial, yeast and human enzymes, the structure of the C-terminal domain has not been determined, though it has been suggested to form a beta propeller form. The holoenzyme has not been crystallized, so its proposed structure is reliant on cryo EM images. The domain structure of human topoisomerase enzymes is outlined in Figure 1.

Eukaryotic type II topoisomerases are dimers that can introduce two transient DNA breaks with a 4 bp stagger in a single DNA duplex, producing a protein bound DNA double strand break or gate. A second DNA duplex termed a ‘transported’ or ‘T’ segment can be captured by the N-terminal domain of the protein, and passed through this transient break in a ‘gate’ or ‘G’ segment. The protein associated DNA break is subsequently resealed [8]. Two DNA interaction surfaces have been proposed. Evidence for one site, consisting of a pair of semicircular grooves on the B' and A' subdomains that are thought to bind to ‘G’ segment, has been reported following footprinting experiments and structural work [9]–[10]. The cleavage reaction is carried out via an active site tyrosine residue located in the core of the enzyme, with the cleaved ‘G’ segment interacting with a helix turn helix (HTH) motif in the CAP domain of the core [11]. In particular, the α4 ‘recognition helix’ has been shown to interact with substrate DNA [12]. A recent crystal structure of *S. cerevisiae* topoisomerase II with DNA showed that on binding the ‘G’ segment the enzyme induces a 150° bend in the DNA and undergoes large conformational changes such that an essential metal ion is positioned near to the reactive tyrosine ready for cleavage [13]. Additionally, a second site has been proposed in the N-terminal half of the enzyme, which could potentially interact with the ‘T’ segment, following the discovery of a groove lined with arginines in the crystal structure of *E. coli* GyrB [14].

Metal ions, in particular divalent cations, are essential in the mechanism of topoisomerase II. Magnesium is required for stabilising the interaction with ATP in the N-terminal domain. The phosphodiester cleavage reaction by a tyrosine in the enzyme core also requires divalent metal ions and thus metal ions are required to support strand passage. Experiments with eukaryotic *D. Melanogaster* topoisomerase II reported that only Mg^2+^ could support strand passage, in contrast to subsequent work with *E. coli* gyrase which showed that Mg^2+^, Ca^2+^, Mn^2+^ and Co^2+^ could all support activity [15]–[16], thus the interaction with metal ions differs in topoisomerase II isoforms from different species.

Three very conserved motifs (EDGSA, IMTDQ and PLRGK) are important for type II topoisomerase function [17]. Altered magnesium optima were seen in proteins mutated within these motifs in human topoisomerase IIβ and two possible binding sites were proposed for the metal ion(s) needed for DNA cleavage [18]. Subsequently, a two metal ion mechanism analogous to DNA polymerases was proposed for the cleavage reaction [15], [18]–[19]. A recent crystal structure of the central breakage reunion domain from yeast with DNA in a cleavage competent conformation has two metal ions present, but these are zinc not magnesium [20]. In contrast other crystal structures of core domains of type II topoisomerases show only one metal ion [21]–[22]. Work with human topoisomerase IIα and human topoisomerase IIβ indicated that interactions with the second metal ion may enhance the ability of topoisomerase IIβ, but not topoisomerase IIα to cleave DNA [23]. Whether one or two ions are needed for cleavage, and which ions these are, requires further study. In addition to a catalytic role, metal ions have also been reported to play a structural role in type II topoisomerase [24]–[25].

Eukaryotic type II topoisomerases have been shown, in strand passage experiments, to prefer supercoiled substrates over relaxed ones, with selectivity for crossovers, both experimentally via electron microscopy experiments, and *in silico*. Such selectivity may represent a mechanism for topological recognition [26]–[28].

Relaxation of supercoiled plasmid DNA by topoisomerases occurs via a strand passage reaction that can produce a series of topoisomers, with differing levels of superhelicity. This pattern may be either processive, in which case the protein stays on the same DNA molecule to relax it further, or distributive, where the enzyme moves onto another supercoiled DNA molecule after one or more rounds of relaxation. Previously, it was reported that topoisomerase IIα gave a more distributive pattern in relaxation assays, while topoisomerase IIβ gave a highly processive pattern [29], consistent with subsequent work showing that topoisomerase IIα and topoisomerase IIβ interact differently with supercoiled substrates. Topoisomerase IIα had a 10-fold preference for positive over negative supercoils whereas topoisomerase IIβ showed no preference [30]. Experiments with C-terminal domain swapped proteins suggested that this geometry sensing was linked to the C-terminal domain of the enzymes [31]. Consistent with this, experiments show topoisomerase II from Paramecium bursaria chlorella virus (PBCV) or chlorella virus Marburg-1 (CVM-1), whose N-terminal and core domains have high homology to eukaryotic topoisomerase II but which naturally lack the C-terminal domain, have no preference for positive or negative supercoils in relaxation [32]. We have previously shown that the C-terminal domain of human topoisomerase IIα and topoisomerase IIβ affects levels of strand passage *in vitro* as well as levels of cell growth in complementation analysis, and suggested that the topoisomerase IIβ C-terminal domain may act as a negative regulator [33].

In this study we have examined the role of the C-terminal domain in the topoisomerase II-DNA interaction using fluorescence anisotropy. We have also examined the role of divalent metal ions in strand passage activity and in DNA binding. We find that the strength of interaction of human topoisomerase IIβ, but not topoisomerase IIα, with a DNA substrate is significantly increased in the absence of the C-terminal domain, suggesting that the C-terminal domain of topoisomerase IIβ acts as a negative regulator. Additionally, we find that the supporting metal ion has no impact on the binding of DNA. The strand passage reaction with both human topoisomerase II enzymes can be supported by calcium and manganese ions in addition to magnesium ions, in contrast to the reported activity of Drosophila topoisomerase II.

## Materials and Methods

### Materials

Oligonucleotides were purchased from MWG and had the following sequence: forward – 5′-CGCAATCTGACAATGCGCTCATCGTCATCCTCGCGACGCG-3′, reverse – 5′-CGCGTGCCGAGGATGACGATGAGCGCATTGTCAGATTGCG-3′. kDNA was purchased from TopoGEN (Columbus, OH).

### Protein Preparation

Proteins were overexpressed and purified from *S.cerevisiae* strain JEL1Δtop1 as described previously [34]–[35]. Full length topoisomerase IIα was expressed from plasmid YEpWob6 [36] and full length topoisomerase IIβ was expressed from plasmid YEphTOP2βKLM [37]. Truncated topoisomerase IIα and truncated topoisomerase IIβ were expressed from plasmids YEphTOP2αt(1242) and YEphTOP2βt(1263) respectively, as we reported previously [33]. For anisotropy experiments, proteins were dialysed into 50 mM Tris pH 8, 10% glycerol, 1 mM EDTA, 1 mM EGTA, 150 mM KCl with protease inhibitors.

### *In vitro* activity assays

Decatenation assays were performed as described previously [35], [38], using 400 ng kDNA. Reactions were performed in ‘relaxation buffer’ (50 mM Tris-HCl pH 7.5, 10 mM MgCl_2_, 0.5 mM EDTA, 30 µg/mL bovine serum albumin (BSA), 1 mM DTT, 100 mM KCl). To assess the impact of different metals, 10 mM MgCl_2_ was replaced by 10 mM CaCl_2_, 10 mM MnCl_2_, 10 mM NiCl_2_ or 10 mM CoSO_4_ as appropriate. In all cases reaction products were quantified using TINA version 2.09d densitometry software. The ratio of decatenated DNA to the total DNA was measured in each case and compared to a control lane. Statistical significance was assessed using a one-sample t-test measuring variance from 100%, with p<0.05 considered significant.

### DNA-binding measurements using fluorescence anisotropy

DNA binding capacity was determined with purified protein and a hexachlorofluorescein (HEX) labelled 40 bp double stranded DNA oligo using fluorescence anisotropy. Measurements were carried out at 20°C using a SLM-Amnico 8100 spectrofluorometer (SLM-Amnico, Urbana, IL). The excitation wavelength was 530 nm with an excitation slit width of 8 mm and the emission wavelength was 570 nm with an emission slit width of 3 mm. A 1 ml fluorescence cuvette was used with excitation and emission paths each of 10 mm. Assays were carried out in anisotropy buffer (50 mM Tris pH 8, 5% glycerol, 50 mM KCl, 1% Triton X-100) supplemented with 100 µg/ml acetylated BSA, and topoisomerase II proteins were matched for buffers and salt concentration. 1 µM HEX-labelled oligo was added to the buffer and a baseline reading taken. Protein was added and an average anisotropy of 12 readings over 99 seconds measured for each titration point. 10 mM MgCl_2_ or 10 mM CaCl_2_ was added to the buffer where described. A one-binding site hyperbola was fitted to data and the Bmax and K_D_ calculated using GraphPad Prism 5. Statistical significance was assessed using an unpaired, two tailed Student's t-test, with p<0.05 considered significant.

## Results

### The effect of the C-terminal domain on DNA binding

The binding of both full length and C-terminally truncated human recombinant DNA topoisomerase IIα and β enzymes to DNA was measured using fluorescence anisotropy, a method we have previously used to assess binding between enzyme and a 40 bp oligo with an mAMSA binding site [35].

**Figure 2:**
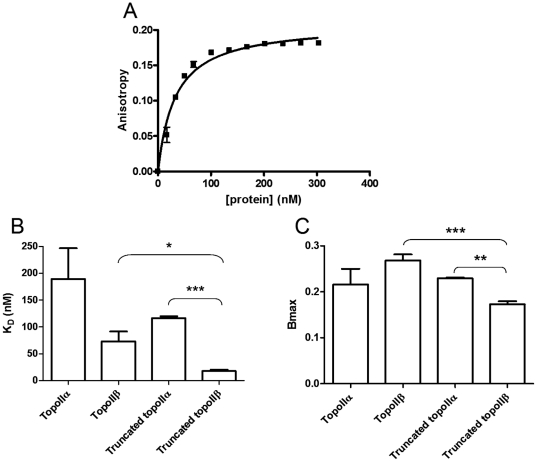
Binding of full length and C-terminally truncated topoIIα and topoIIβ to a 40 bp DNA oligo. (**A**) Typical anisotropy curve, measuring topoisomerase IIβ binding to oligo AB. (**B**) K_D_ of binding (in nM). (**C**) Maximum anisotropy (Bmax). The average of at least three independent experiments is shown with error bars representing standard error from the mean. Statistical significance is indicated with * representing p<0.05, ** representing p<0.01 and *** representing p<0.001.

Fluorescence anisotropy can be used to measure the interaction between two molecules and to derive a binding constant. The two molecules used here are an oligonucleotide labelled with a hexafluorescein tag and purified unlabelled recombinant human type II topoisomerases. When a complex forms between the oligonucleotide and protein it changes the environment of both macromolecules, and alters the rate at which the molecules tumble in solution. Excitation of the fluorophore by polarised light enables it to emit polarised light of a different wavelength, but if the molecule is tumbling free in solution the emitted light radiates in different directions and the light signal is scrambled rather than polarised. If the oligonucleotide bearing the fluorophore binds to a protein it will tumble less, reducing the scrambling and enabling more polarised light to be detected. When all the fluorophore is bound to protein the binding curve plateaus indicating that the maximum anisotropy or maximum binding (Bmax) has been reached. Differences in Bmax indicate a difference in binding mode, although specific mechanistic details cannot be inferred from this data. The K_D_ is the concentration of ligand required to give half maximal binding. An example plot is shown in Figure 2a. Measurements were determined as described in Materials and Methods.

Four different proteins were used, full length topoisomerase IIα, full length topoisomerase IIβ, and each isoform without its C-terminal domain, referred to as truncated topoisomerase IIα and truncated topoisomerase IIβ. A minimum of three independent experiments was done for each enzyme variant; for each protein concentration 12 readings were taken over 99 seconds and averaged. The average anisotropy readings were plotted versus protein concentration, and the resultant curves analysed to give dissociation constants (K_D_) and maximum anisotropy indicating the maximal binding (Bmax). The measured values are shown in Table 1 and Figure 2.

**Table 1:** Binding of full length and C-terminally truncated topoIIα and topoIIβ to a 40 bp DNA oligo, showing binding affinity (K_D_) and maximum anisotropy (Bmax).

	K_D_ (nM)	Bmax
Topoisomerase IIα	189±57.4	0.2156±0.034
Topoisomerase IIβ	72.9±18.6	0.2679±0.013
Truncated topoisomerase IIα	116.3±3.48	0.2292±0.0016
Truncated topoisomerase IIβ	17.8±2.72	0.1726±0.0069

The average of at least three independent experiments is shown with standard error from the mean indicated.

The K_D_ of full length topoisomerase IIβ was found to 72.9 nM which while lower than that of full length topoisomerase IIα at 189 nM (implying stronger binding), is not a significant difference, with a p value of 0.0542 by Student's t-test. There was no significant difference in binding strength between the full length and truncated versions of topoisomerase IIα. The difference in K_D_ between truncated topoisomerase IIβ and the other isoforms is more striking, with truncated topoisomerase IIβ having a K_D_ of 18 nM, showing more than 6 times stronger binding than truncated topoisomerase IIα which has a K_D_ of 116 nM (p<0.0001). Additionally, truncated topoisomerase IIβ gave significantly stronger binding than the full length version, with a p value of 0.0189 by Student's t-test. Our unpublished data with 3 other 40 bp oligonucleotides and all four proteins confirmed that truncated topoisomerase IIβ bound most strongly.

No significant difference was seen in maximal binding for the full length topoisomerase IIα, the full length topoisomerase IIβ or the C-terminally truncated topoisomerase IIα, their Bmax values being 0.215, 0.268 and 0.229 respectively. However the Bmax of the truncated topoisomerase IIβ at 0.173 was significantly lower than either the full length topoisomerase IIβ (p = 0.0002) or the truncated topoisomerase IIα (p = 0.0045), suggesting that the lack of the topoisomerase IIβ C-terminal domain significantly alters the maximal binding of this protein. While it is not possible to ascertain specific mechanistic details from this data, the lower Bmax of truncated topoisomerase IIβ indicates that this enzyme has a different mode of binding to the DNA substrate, in addition to the stronger binding shown by lower K_D_, when compared with the other isoforms.

### Strand Passage of human topoisomerase II with different metals

**Figure 3:**
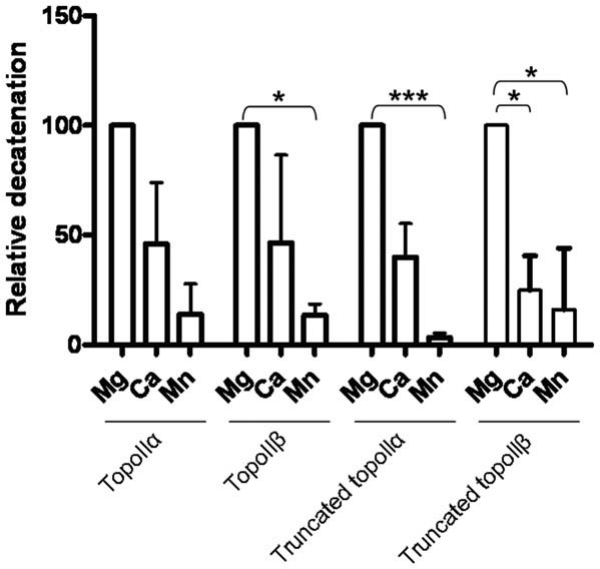
Relative decatenation of full length and C-terminally truncated topoIIα or topoIIβ with different supporting metal ions. Mg – Magnesium, Ca – Calcium, Mn – Manganese. For each protein, strand passage with magnesium ions was taken as 100% and other values calculated relative to this. The average of at least two independent experiments is shown, with error bars representing standard error from the mean. Statistical significance is indicated with * representing p<0.05 and *** representing p<0.001.

Previous work with *E. coli* gyrase has shown that Ca^2+^, Mn^2+^ and Co^2+^, in addition to Mg^2+^, can support the strand passage reaction [15], in contrast to *D. Melanogaster* topoisomerase II where only Mg^2+^ could support strand passage [16], indicating that there may be species specific differences in this regard. Strand passage by full length and C-terminally truncated topoisomerase IIα and topoisomerase IIβ was investigated using Mg^2+^, Ca^2+^, Mn^2+^, Co^2+^ and Ni^2+^ as potential supporting ions. For all four proteins the degree of strand passage varied with supporting ion. Figure 3 and Table 2 show the average decatenation of full length and truncated isoforms in the presence of magnesium, calcium or manganese. In each case decatenation with magnesium was set to 100% and that with calcium and manganese is expressed relative to this. In all cases the preference of ions for strand passage was similar; greatest decatenation was found with magnesium, then calcium, and then manganese, an order that is consistent with the results reported with *E. coli* gyrase [15]. Nickel and cobalt supported no detectable strand passage under the conditions used here.

**Table 2:** Relative decatenation of full length or C-terminally truncated topoIIα or topoIIβ with magnesium (Mg^2+^), Calcium (Ca^2+^) or Manganese (Mn^2+^) as supporting ion.

	Mg^2+^	Ca^2+^	Mn^2+^
Topoisomerase IIα	100	46.12±27.9	13.91±13.91
Topoisomerase IIβ	100	46.59±40.02	13.59±5.16
Truncated topoisomerase IIα	100	40.07±15.25	3.41±2.08
Truncated topoisomerase IIβ	100	24.9±9.05	16.14±16.14

For each protein, strand passage with magnesium ions was taken as 100% and other values calculated relative to this. The average of at least two independent experiments is shown, with standard error from the mean indicated.

The significance of the degree of activity relative to that with magnesium was assessed using a one-sample t-test measuring variance from 100%. Manganese supported significantly less decatenation than that of magnesium when topoisomerase IIβ (p = 0.0379), truncated topoisomerase IIα (p = 0.0005) and truncated topoisomerase IIβ (p = 0.0351) were considered. Considering calcium, only truncated topoisomerase IIβ gave significantly less decatenation than with magnesium (p = 0.0142). There was no significant difference in the relative response to metals by isoform or upon removing the C-terminal domain.

### DNA binding to 40 bp oligo in the presence of metal ions

The level of strand passage by topoisomerase IIα and topoisomerase IIβ varies significantly with supporting metal ion (above). To determine whether this was due to differences in interaction strength with DNA, the interaction of human topoisomerase IIα or topoisomerase IIβ with the 40 bp DNA oligo described above, in the absence of ions as well as the presence of magnesium or calcium ions, was assessed.

**Figure 4:**
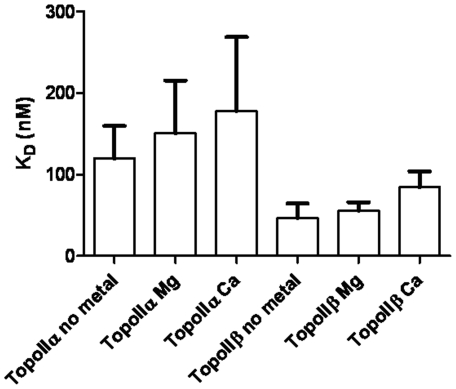
The K_D_ of binding to oligo AB (in nM) of full length topoIIα and topoIIβ, in the absence of metal or the presence of magnesium (Mg) or calcium (Ca) ions. The average of at least two independent experiments is shown, with error bars representing standard error from the mean.

Data are shown in Figure 4 and Table 3. The DNA binding affinity of neither topoisomerase IIα nor topoisomerase IIβ is significantly altered by the presence of magnesium or calcium, as seen previously by gel mobility shift assay [39]. As above, in all cases the topoisomerase IIα protein binds to the oligo less strongly than the topoisomerase IIβ protein. In the absence of metal ions, topoisomerase IIα binds with an affinity of 160.5±49.7 nM, while topoisomerase IIβ binds with an affinity of 46.4±18.1 nM, a difference that is statistically significant (p = 0.0276). In the presence of magnesium ions topoisomerase IIα binds with an affinity of 150.3±65.0 nM, and topoisomerase IIβ with an affinity of 55.4±10.7 nM, a difference that is also statistically significant (p = 0.0401). While topoisomerase IIα binds less strongly, again, than topoisomerase IIβ in the presence of calcium ions, with K_D_s of 177.7±90.9 and 84.3±19.5 respectively (p = 0.2867), the difference did not reach significance with 3 replicates.

**Table 3:** The K_D_ of binding of topoIIα or topoIIβ (in nM) to DNA with no metal, 10 mM MgCl_2_, or 10 mM CaCl_2_.

	K_D_ TopoIIα (nM)	K_D_ TopoIIβ (nM)
No metal	160.5±49.66	46.35±18.09
10 mM MgCl_2_	150.3±65.01	55.39±10.7
10 mM CaCl_2_	177.7±90.89	84.33±19.45

The average of at least two independent experiments is shown with standard error of the mean indicated.

## Discussion

We have used fluorescence anisotropy to investigate the DNA interaction between the two human topoisomerase II isoforms, and the impact of the C-terminal domain on this interaction. Firstly, the relative binding affinities of topoisomerase IIα and β, both full length and C-terminally truncated, were assessed using a 40 bp oligo with an mAMSA cleavage site that has been used previously in DNA binding studies [35], [39]–[40]. The K_D_ of binding determined by fluorescence anisotropy was comparable to that found by gel mobility shift analysis on the same oligonucleotide substrate (130 nM) [39], but differed from that found by surface plasmon resonance (SPR – 1.73 nM) [40]. SPR differs from the first two assays in that the DNA substrate is fixed a one end, so reducing its ability to diffuse away from the protein, which may account for the difference in K_D_ seen.

Topoisomerase IIβ, whether full length or C-terminally truncated, gave stronger binding than its topoisomerase IIα counterpart, suggesting topoisomerase IIβ binds DNA with higher affinity, consistent with isoform specific DNA interactions [29]–[30]. The anisotropy data is most striking when the truncated topoisomerase IIβ isoform is considered, this having significantly lower maximum anisotropy than the other three isoforms. When three other oligonucleotides were tested truncated topoisomerase IIβ had the strongest binding of all four enzyme isoforms (unpublished data). The significantly stronger binding of topoisomerase IIβ once the C terminal domain is removed supports the idea that the C-terminal domain may act as a negative regulator of DNA interaction, and that this could provide a rationale for isoform specific functions in vivo [33].

Metal ions are needed in the strand passage reaction both to coordinate ATP for hydrolysis and to polarise the active site tyrosine before the cleavage reaction. Work reported previously indicated that *D. Melanogaster* topoisomerase II could only perform strand passage in the presence of magnesium ions [16], in contrast to work with bacterial gyrase which showed that magnesium, calcium, manganese and cobalt ions could all support strand passage [15]. We show that magnesium, calcium and manganese ions can all support the strand passage reaction in human topoisomerase IIs, to varying degrees, in an order consistent with previous work [15]. We found no evidence that cobalt ions could support the strand passage reaction of human topoisomerase IIs, in contrast to an earlier report [41]. The reason for this difference is unknown, but could be related to the metal salt forms used – Baldwin et al. use the chloride salt form, whereas the sulphate form was used in this case. Interestingly cobalt, but not magnesium, calcium or manganese, supported cleavage of the topoisomerase IIβ enzyme using a metal affinity cleavage assay based on Fenton chemistry [42] (unpublished data). No cobalt was found to be associated with either topoisomerase IIα or IIβ when an elemental profile was established using Inductively Coupled Plasma Mass Spectrometry (ICP-MS), although interestingly zinc was found to be consistently associated with both isoforms using this technique (unpublished data).

The strength of interaction of topoisomerase IIα or topoisomerase IIβ with DNA wasn't affected by the presence of magnesium or calcium ions, indicating that the difference in the level of strand passage supported by different ions may be due to differences in their ability to support the phosphoryl-transfer reaction of cleavage, rather than differences in DNA substrate interaction. The presence or absence of the C-terminal domain of either isoform had no effect on the relative levels of strand passage with each protein, indicating that the C-terminal domain is not involved in the co-ordination of metal ions for strand passage. This is consistent with the proposed location of the metal ion binding sites in the B' region of the core of the enzyme [15], [18]–[19], and recent topoisomerase II-DNA structures [20]–[22], [24].

In conclusion, we have shown that the C-terminal domain of topoisomerase IIβ, but not topoisomerase IIα, has a significant effect on the K_D_ with DNA, providing further evidence that this region of topoisomerase IIβ may have a negative regulatory role [33].
